# Targeting Mitochondrial Dysfunction to Prevent Endothelial Dysfunction and Atherosclerosis in Diabetes: Focus on the Novel Uncoupler BAM15

**DOI:** 10.3390/ijms26104603

**Published:** 2025-05-11

**Authors:** Woong Bi Jang, Vinoth Kumar Rethineswaran, Sang-Mo Kwon

**Affiliations:** 1 Laboratory for Vascular Medicine and Stem Cell Biology, Department of Physiology, Medical Research Institute, School of Medicine, Pusan National University, Yangsan 50612, Republic of Korea; jangwoongbi@naver.com (W.B.J.); vinrebha@gmail.com (V.K.R.); 2 Convergence Stem Cell Research Center, Pusan National University, Yangsan 50612, Republic of Korea

**Keywords:** diabetes mellitus, endothelial dysfunction, atherosclerosis, mitochondrial dysfunction, mitochondrial uncoupling

## Abstract

Diabetes mellitus is a chronic metabolic disorder characterized by persistent hyperglycemia, leading to endothelial dysfunction and accelerated atherosclerosis. Mitochondrial dysfunction, oxidative stress, and dysregulated lipid metabolism contribute to endothelial cell (EC) injury, promoting plaque formation and increasing cardiovascular disease risk. Current lipid-lowering therapies have limited effectiveness in restoring endothelial function, highlighting the need for novel strategies. Mitochondrial uncoupling has emerged as a promising approach, with BAM15—a newly identified mitochondrial uncoupler—showing potential therapeutic benefits. BAM15 enhances fatty acid oxidation (FAO), reduces reactive oxygen species, and protects ECs from hyperglycemia-induced apoptosis. Unlike conventional uncouplers, BAM15 demonstrates improved tolerability and efficacy without severe off-target effects. It restores mitochondrial function, improves endothelial survival, and supports metabolic homeostasis under hyperglycemic conditions. This review uniquely integrates emerging evidence on mitochondrial dysfunction, endothelial metabolism, and FAO to highlight the novel role of BAM15 in restoring vascular function in diabetes. We provide the first focused synthesis of BAM15’s mechanistic impact on EC bioenergetics and position it within the broader landscape of mitochondrial-targeted therapies for diabetic vascular complications. Further research is needed to elucidate the molecular mechanism through which BAM15 modulates EC metabolism and to evaluate its long-term vascular effects in diabetic models.

## 1. Introduction

Diabetes mellitus (DM) is a chronic metabolic disorder marked by persistent hyperglycemia from insulin deficiency, resistance, or both [1,2,3]. According to the International Diabetes Federation, 537 million people had diabetes in 2021, a number projected to rise to 643 million by 2030 and 783 million by 2045 [4]. During this period, while the global population is expected to rise by 20%, diabetic cases may grow by 46% [4]. DM is classified into three types: type 1 diabetes (T1D), type 2 diabetes (T2D), and gestational diabetes [5,6,7]. T1D is an autoimmune condition leading to the destruction of pancreatic beta-cells, accounting for 5 to 10% of cases, and is influenced by genetic, autoimmune, and environmental factors [8,9]. T2D represents 90% to 95% of all cases and is commonly associated with obesity and physical inactivity, leading to insulin resistance and an eventual reduction in insulin production [10,11,12,13]. Diabetes and its complications remain a major cause of global morbidity and mortality.

While classified as a metabolic disorder, diabetes is fundamentally a vascular disease due to its significant impact on both microvascular and macrovascular systems. Microvascular complications—such as diabetic retinopathy, neuropathy, and nephropathy—contribute to substantial long-term morbidity [14,15]. Macrovascular complications manifest as accelerated atherosclerosis, leading to peripheral vascular disease, early coronary artery disease (CAD), and an increased risk of cerebrovascular events [16,17,18]. Diabetes impacts the vascular wall through endothelial dysfunction, oxidative stress, and inflammation [19,20]. This review examines the complex interplay between diabetes, endothelial dysfunction, and atherosclerosis, emphasizing mitochondrial dysfunction as a key pathological driver. We discuss mitochondrial uncoupling as a novel therapeutic strategy to mitigate diabetes-induced vascular damage, with a focus on BAM15—a mitochondrial uncoupler shown to restore endothelial function and prevent atherosclerotic plaque formation. By enhancing fatty acid oxidation (FAO) and reducing oxidative stress, BAM15 offers a promising approach for treating diabetes-associated atherosclerosis.

## 2. Endothelial Dysfunction and Atherosclerosis in Diabetes: The Role of Nitric Oxide, Insulin Signaling, and Metabolic Dysregulation

Blood vessel walls consist of three concentric layers: the intima, medium, and adventitia. The intima contains a thin layer of endothelial cells (ECs) [21]. The endothelium, a monolayer of ECs approximately ≤1 μm thick, spans a surface area of 4000–7000 m^2^. It functions as a structural barrier between the vessel lumen and wall and serves critical autocrine and paracrine roles. The endothelium produces various growth factors and cytokines that regulate key vascular functions, including vascular tone, smooth muscle cell proliferation, platelet aggregation, coagulation, and fibrinolysis [22]. ECs play a central role in maintaining vascular homeostasis by balancing the production of vasodilators and vasoconstrictors. Vasoconstriction is mediated by factors such as endothelin-1 (ET-1) and thromboxane A2, whereas vasodilation is regulated by nitric oxide (NO), prostacyclin, and endothelial-derived hyperpolarizing factor (EDHF) [22,23]. Blood fluidity and coagulation are controlled through the regulation of platelet activity, the coagulation cascade, and the fibrinolytic system. Inflammatory responses are modulated by cytokines and adhesion molecules expressed by ECs. Endothelial dysfunction—characterized by impaired barrier function, reduced vasodilation, and disruptions in proliferation, migration, tube formation, and angiogenesis—is commonly observed in individuals with cardiovascular disease (CVD), insulin resistance, obesity, and diabetes.

### 2.1. Impaired Nitric Oxide Signaling in Endothelial Cells: A Key Contributor to Vascular Dysfunction

**Figure 1:**
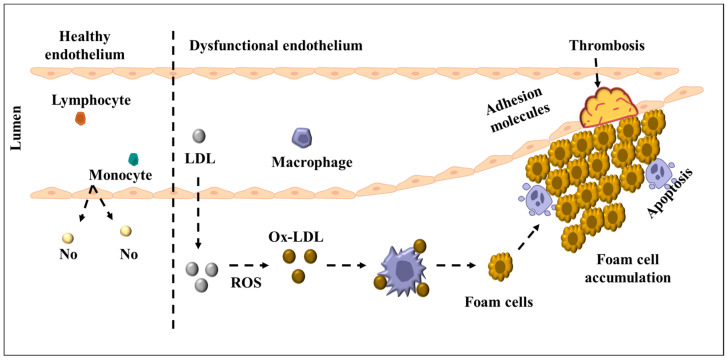
Endothelial cells (ECs) facilitate low-density lipoprotein (LDL) transcytosis into the subendothelial space, initiating a vascular inflammatory response. In early atherosclerosis, the endothelium shifts from a quiescent to activated state in response to pro-atherogenic stimuli such as oxidized LDL (ox-LDL), pro-inflammatory cytokines, and disturbed flow. Activated ECs recruit immune cells, including T lymphocytes, neutrophils, and monocytes, into the intima. Monocyte-derived macrophages engulf lipids to form foam cells, which undergo necrosis and apoptosis, contributing to the lipid core of growing plaques. Increased endothelial permeability further facilitates LDL entry into the arterial wall, a key early event in plaque initiation, progression, and eventual rupture.

NO is a key signaling molecule produced by ECs, essential for maintaining vascular homeostasis through the regulation of vasodilation, inhibition of thrombosis, and reduction in inflammation [24,25]. It is synthesized by endothelial nitric oxide synthase (eNOS), which converts L-arginine into NO and citrulline. eNOS activity is tightly regulated by intracellular factors, including calcium/calmodulin signaling and protein interactions [26,27,28]. A major regulator is caveolin-1, a structural protein found in caveolae—cholesterol-rich invaginations of the EC membrane. Under resting conditions, caveolin-1 binds to eNOS, maintaining it in an inactive state. eNOS activation requires an increase in intracellular calcium, which promotes the binding of calcium ions (Ca^2^⁺) to calmodulin. This interaction induces a conformational change in eNOS, displacing caveolin-1 and activating eNOS [29,30,31,32]. Once active, eNOS produces NO, which diffuses into adjacent vascular smooth muscle cells (VSMCs) and activates soluble guanylate cyclase (sGC). This enzyme converts guanosine triphosphate (GTP) into cyclic guanosine monophosphate (cGMP), a secondary messenger that promotes smooth muscle relaxation and vasodilation. This mechanism is fundamental to the maintenance of normal blood flow and blood pressure regulation [33,34,35,36,37]. Endothelium-derived NO is essential for counteracting vasoconstrictive forces within the vascular system. NO inhibits the effects of ET-1 and angiotensin II (Ang II), both potent vasoconstrictors. By maintaining vascular tone, NO prevents the excessive constriction of blood vessels, ensuring adequate tissue and organ perfusion. Additionally, NO exerts multiple anti-atherogenic effects, preventing platelet adhesion and aggregation, reducing the likelihood of thrombosis. Furthermore, NO inhibits the adhesion and infiltration of leukocytes into the vascular endothelium, a key step in the initiation of atherosclerosis. NO also suppresses the proliferation and migration of vascular smooth muscle cells, which contribute to the formation of atherosclerotic plaques [23,38]. Despite its protective functions, NO signaling can be severely disrupted by oxidative stress, leading to endothelial dysfunction. The increased production of reactive oxygen species (ROS), such as superoxide anions (O_2_^−^) and peroxynitrite (ONOO^−^), can scavenge NO, effectively reducing its bioavailability. The reduction in NO levels leads to increased vasoconstriction, impaired vasorelaxation, and a pro-inflammatory state within the endothelium. A major consequence of oxidative stress is the oxidation of low-density lipoprotein (LDL) cholesterol, which plays a pivotal role in the development of atherosclerosis [38,39]. Oxidized LDL (oxLDL) exerts multiple deleterious effects on endothelial NO signaling, a process illustrated in Figure 1. It has been shown to upregulate caveolin-1 expression, leading to the increased sequestration and inactivation of eNOS, thereby reducing NO production. Additionally, oxLDL disrupts the structural integrity of caveolae, where eNOS is predominantly localized. The loss of caveolae integrity is associated with decreased eNOS activity and impaired endothelial function. Furthermore, oxLDL promotes a pro-inflammatory response by stimulating the expression of adhesion molecules, such as vascular cell adhesion molecule-1 (VCAM-1), intercellular adhesion molecule-1 (ICAM-1), and E-selectin, which facilitate leukocyte adhesion and infiltration into the vascular wall (Figure 1). This process exacerbates endothelial dysfunction, promoting the initiation and progression of atherosclerosis [40,41]. oxLDL cholesterol stimulates caveolin-1 production, which reduces NO generation by inactivating eNOS [42]. In experimental models, the exposure of ECs to oxLDL results in the disruption of the caveolae–eNOS complex, leading to reduced NO synthesis and an increased susceptibility to vasoconstrictive stimuli such as angiotensin II and ET-1. In vivo studies have further demonstrated that reduced NO bioavailability is associated with increased arterial stiffness, heightened vascular inflammation, and enhanced oxidative stress, all of which contribute to the pathophysiology of CVD [43]. Collectively, impaired NO signaling in ECs is a key driver of vascular dysfunction. Whether through increased caveolin-1-mediated inhibition of eNOS, oxidative stress-induced NO depletion, or oxLDL-mediated disruption of caveolae, the loss of NO bioavailability has profound implications for vascular health. Given its critical role in maintaining endothelial function, strategies aimed at preserving NO signaling—such as antioxidant therapy, caveolin-1 inhibition, or enhancing eNOS activity—could provide therapeutic benefits in preventing or mitigating the progression of atherosclerosis and other CVDs [44].

### 2.2. Disrupted Insulin Signaling in Endothelial Cells and Its Impact on Vascular Homeostasis

EC dysfunction is a hallmark of vascular diseases and is driven by several risk factors, including hypertension, DM, hypercholesterolemia, obesity, chronic hyperglycemia, advanced glycation end products (AGEs), and genetic predispositions. Among these, prolonged exposure to elevated blood glucose is a primary contributor to endothelial impairment. Chronic hyperglycemia induces metabolic and structural changes in ECs, impairing their ability to regulate vascular tone, maintain barrier integrity, and control inflammatory responses [45]. A key mediator of glucose metabolism in ECs is glucose transporter 1 (GLUT1), the most abundant glucose transporter in these cells [46]. Initially, GLUT1 was thought to be insensitive to changes in glucose levels; however, more recent studies indicate that prolonged exposure to high glucose concentrations downregulates GLUT1 expression and reduces the rate of glucose uptake [47]. By limiting glucose absorption, these alterations in GLUT1 expression may protect ECs from the injury induced by high glucose intake. Excess AGEs cause endothelial dysfunction during diabetes by binding to the receptor for AGEs, resulting in increased EC permeability, the suppression of eNOS activity, and coagulation system impairment [48]. Hyperglycemia-induced endothelial dysfunction is further exacerbated by oxidative stress. Chronic high glucose levels stimulate excessive mitochondrial ROS production, which contributes to the inhibition of NO synthase (NOS) and enhances the production of pro-inflammatory cytokines, chemokines, and adhesion molecules. These changes lead to increased leukocyte adhesion and infiltration, which accelerates the inflammatory response in the vascular endothelium, elevated platelet aggregation, contributing to pro-thrombotic conditions, and mitochondrial dysfunction, further amplifying ROS generation and cellular damage [49]. Beyond hyperglycemia, insulin resistance and impaired insulin signaling also play a key role in endothelial dysfunction. Insulin resistance—defined by reduced cellular responsiveness to insulin—leads to hyperglycemia, hyperinsulinemia, and dyslipidemia, all of which contribute to vascular injury. Under normal physiological conditions, insulin signaling in ECs is vital for vascular homeostasis, regulating the release of vasoactive molecules, and maintains the balance between vasodilation and vasoconstriction [50]. Insulin primarily exerts its vascular effects through interaction with the insulin receptor and insulin-like growth factor-1 receptor. The activation of these receptors initiates intracellular signaling pathways involving in glucose uptake, protein synthesis, lipid metabolism, and glycogen storage. Importantly, insulin also stimulates eNOS activation, leading to increased NO production and enhanced endothelium-dependent vasodilation [11]. However, in insulin-resistant states, this pro-vasodilatory insulin signaling is impaired, while pro-atherogenic signaling pathways remain active. Specifically, insulin resistance leads to reduced eNOS activation, diminishing NO production and endothelial function, increased ET-1 production, promoting vasoconstriction and hypertension, excessive ROS generation, further suppressing NO bioavailability and worsening oxidative stress, and dysregulated lipid metabolism, leading to higher circulating free fatty acids (FFAs), which induce endothelial inflammation and apoptosis.

**Figure 2:**
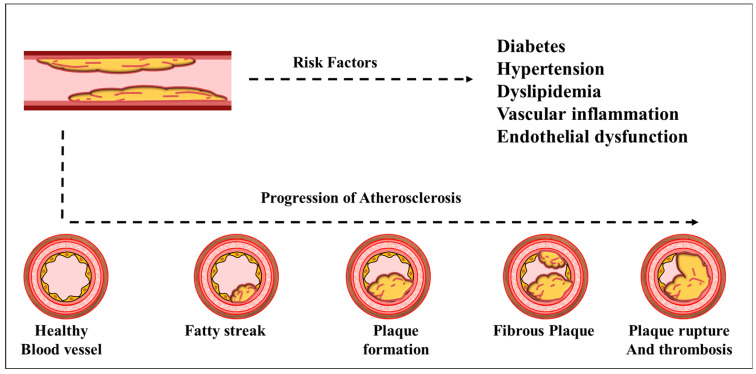
Schematic of atherosclerotic plaque formation from a healthy artery to plaque rupture, highlighting the most critical events that lead to its development at each stage. Diabetes, hypertension, dyslipidemia, vascular inflammation, and endothelial dysfunctions are all variables that contribute to plaque formation and rupture. Atherosclerosis develops gradually as cholesterol, fat, blood cells, and other blood components combine to produce plaque. When plaque builds up in the arteries, it causes them to narrow. This reduces the transport of oxygen-rich blood to tissues and vital organs in the body.

Beyond its role in glucose homeostasis, insulin is crucial for regulating protein turnover, post-translational modifications, and intracellular signaling pathways. Disrupted insulin signaling impairs vascular function and promotes atherosclerotic plaque formation by increasing inflammation, endothelial permeability, and thrombosis. This pathological progression from endothelial dysfunction to plaque rupture is illustrated in Figure 2. Given the close link between insulin signaling and endothelial health, restoring insulin sensitivity through lifestyle modifications, pharmacological agents, or targeted molecular therapies offers a promising approach to prevent or reverse vascular complications in individuals with diabetes and metabolic syndrome. In addition to oxidative stress and impaired insulin signaling, monocyte activation plays a critical role in the progression of endothelial dysfunction and atherosclerosis. Under hyperglycemic and insulin-resistant conditions, activated ECs express higher levels of adhesion molecules such as VCAM-1 and ICAM-1, which facilitate the recruitment and adhesion of circulating monocytes to the vascular endothelium [51]. Once transmigrated into the intima, these monocytes differentiate into macrophages and internalize oxLDL, leading to foam cell formation—a hallmark of early atherosclerotic plaque development. The accumulation of foam cells and pro-inflammatory cytokines (e.g., TNF-α, IL-6, MCP-1) further amplifies vascular inflammation and promotes plaque instability [52,53]. This inflammatory cascade is tightly linked to mitochondrial dysfunction and ROS overproduction, which not only perpetuate endothelial damage but also prime monocytes and macrophages for enhanced pro-inflammatory responses [54,55,56,57]. Therefore, targeting both endothelial oxidative stress and monocyte-driven inflammation may offer synergistic benefits in attenuating vascular injury in diabetic patients.

### 2.3. Mitochondrial Dysfunction in Endothelial Cells: A Nexus Between Oxidative Stress and Atherosclerosis

Mitochondrial dysfunction is increasingly recognized as a critical precursor to endothelial dysfunction, particularly in individuals with type 1 and type 2 diabetes [58]. There is accumulating evidence in diabetic research of alterations in mitochondrial shape, as well as a decrease in respiratory chain complex activity and gene expression encoding a crucial enzyme in oxidative metabolism [59]. Surprisingly, all of these processes are connected to increased ROS generation, decreased adenosine triphosphate (ATP) synthesis, and decreased mitochondrial membrane potential. Mitochondria are essential organelles responsible for ATP production through glucose and lipid metabolism [60]. They play a critical role in regulating cell survival and death. Mitochondria provide a significant amount of cellular energy in the form of ATP via mitochondrial complex proteins (I, II, III, IV, and V) found in the inner membrane of the mitochondria. Mitochondrial complex proteins (I, III, and IV) are necessary for the generation of cellular energy during oxidative phosphorylation. The terminal protein in the electron transport chain helps to maintain a low and healthy mitochondrial membrane potential in a relaxed state and prevents the generation of mitochondrial ROS. Impaired complex IV activity in cells leads to a weakened mitochondrial membrane potential, a lower ATP level, and mitochondrial malfunction [60]. There is mounting evidence that T1D and T2D have a major impact on mitochondrial function, ROS production, apoptosis, and EC deaths [61].

Mitochondria are critical for cellular metabolism, energy production, and survival, especially in high-energy-demanding cell types. In addition to energy production, mitochondria are involved in a number of key cellular activities, such as calcium homeostasis and signaling function, which is mostly accomplished through the generation of ROS [62]. The overproduction of ROS in the mitochondria results in mitochondrial damage, the increased oxidation of low-density lipoprotein, and EC dysfunction [63]. A key initiating event in atherosclerotic plaque formation is the oxidation of LDL by ROS and reactive nitrogen species. oxLDL infiltrates the subendothelial space at sites of vascular injury, triggering a strong inflammatory response. This promotes monocyte recruitment, foam cell formation, and plaque destabilization, increasing the risk of atherosclerotic plaque rupture and thrombosis [64]. The oxidation of LDL, which occurs as a result of the interaction of reactive oxygen and RNS, and its transfer into the subendothelial region of the artery wall at areas of endothelial injury, is thought to be an initial event in atherosclerotic plaque initiation, progression, and rupture [65]. While mitochondrial dysfunction has been extensively linked to diabetes-related complications, the precise molecular mechanisms connecting mitochondrial dysfunction to atherosclerosis remain incompletely understood. Further research is necessary to elucidate the complex interplay between mitochondrial bioenergetics, oxidative stress, and vascular pathology, which could provide new therapeutic targets for preventing and treating diabetes-associated vascular diseases and atherosclerosis.

### 2.4. Diabetes-Induced Atherosclerosis: Mechanistic Insights into Lipid Accumulation and Plaque Formation

Atherosclerosis is a progressive vascular disease characterized by the thickening and hardening of the arterial walls due to the accumulation of lipid-rich plaques within the EC layer. The early stage of atherosclerosis is characterized by the formation of fatty streaks, which are formed by lipid-laden foam cells, VSMCs, and T lymphocytes within the vascular wall [65,66]. If endothelial damage persists, these fatty streaks can progress into mature atherosclerotic plaques. Atherosclerotic lesions are hypothesized to develop as a result of a local inflammation in the artery wall driven by dyslipidemia, namely high LDL cholesterol (LDL-C) and residual lipoprotein levels, as well as other disease variables [67,68]. Oxidized lipoproteins are atherogenic and play a key role in the pathogenesis of coronary heart disease. They have been identified within atherosclerotic lesions in both animal models and human subjects [69]. However, the origin of oxidized lipoproteins in vivo is unknown because the place and process by which lipoproteins oxidize have not been determined. It has been postulated that lipoprotein oxidation may occur locally in the arterial wall or that circulating oxidized lipoproteins are sequestered in atherosclerotic plaques.

Diabetes and dyslipidemia are the primary causes of plaque development that leads to diabetic atherosclerosis. T2D is frequently accompanied by lipid abnormalities, such as increased very-LDL (VLDL) and LDL-C and decreased high-density lipoprotein cholesterol (HDL-C) [68,70]. The glycosylation of extracellular proteins, particularly that of long-lived proteins such as vessel wall collagen, contributes to atherosclerosis by forming AGEs [71]. In diabetes, LDL glycosylation increases with glucose levels, and AGE and Apolipoprotein B levels rise in diabetics [72]. AGEs build regularly on long-lived vessel wall proteins as we age and at a greater rate in diabetes. Clinical research has revealed that diabetics have a higher level of AGEs in their LDL than healthy people [72]. Protein and lipoprotein glycosylation can disrupt normal function by modifying enzyme activity, decreasing degradative capacity, and interfering with receptor binding. The glycosylation process affects both the apoprotein B and the phospholipid components of LDL, resulting in functional changes in LDL clearance as well as increased susceptibility to oxidative modifications [73]. Because of their interaction with proteoglycans, altered LDL particles remain in the subendothelial space at the site of an atherosclerotic lesion for a longer period of time, increasing their chances of being internalized by the lesion cells [74]. Furthermore, because modified LDL has a lower affinity for the LDL receptor (LDLR), it is primarily internalized via unspecific phagocytosis, resulting in intracellular cholesterol accumulation rather than typical lipoprotein particle destruction [75]. These activities result in the formation of foam cells, the cytoplasm of which is densely packed with accumulating lipid droplets. At this point, a lipid core has formed, which will evolve into an atherosclerotic plaque with ongoing input of various inflammatory cell types and extracellular lipids [76]. Diabetes not only accelerates plaque formation but also increases plaque vulnerability, making diabetic patients particularly prone to acute cardiovascular events such as myocardial infarction and stroke. The hypercoagulable state associated with diabetes, driven by increased platelet aggregation and endothelial dysfunction, further contributes to thrombus formation upon plaque rupture. Despite significant progress in understanding diabetes-induced atherogenesis, further research is needed to elucidate the precise molecular mechanisms linking hyperglycemia, lipid metabolism, and vascular inflammation, with the goal of developing more targeted therapeutic interventions to mitigate diabetic cardiovascular complications.

### 2.5. Endothelial Dysfunction as a Precursor to Atherosclerosis: The Role of Chronic Inflammation and Oxidative Stress

Endothelial dysfunction is a hallmark of both diabetes and atherosclerosis, serving as a crucial link between metabolic disorders and CVD. The dysregulation of lipid metabolism in T2D is characterized by hypertriglyceridemia, hyperlipidemia, elevated levels of free fatty acids (FFAs), and an increased proportion of small, dense low-density lipoprotein (sdLDL), all of which contribute to vascular damage and disease progression [77]. Epidemiological and clinical studies have established a strong correlation between elevated circulating triacylglycerol levels and atherosclerosis, reinforcing the notion that lipid overload and endothelial dysfunction are interconnected processes in diabetic vascular complications [78]. ECs are directly affected by circulating lipids such as triglyceride-rich lipoproteins, chylomicron remnants, and FFAs [79]. FFAs are primarily released into the bloodstream via hormone-sensitive lipase activity on stored triacylglycerols in adipose tissue. Additionally, chylomicrons, particularly following high-fat dietary intake, contribute to postprandial plasma FFA elevations. Elevated FFAs in circulation are a major driver of insulin resistance, disrupting insulin signaling pathways within ECs and exacerbating endothelial dysfunction. Insulin-resistant ECs exhibit reduced NO bioavailability, impaired vasodilation, and increased susceptibility to oxidative stress, ultimately setting the stage for atherosclerotic plaque development [80,81]. Endothelial dysfunction extends beyond impaired vasodilation, contributing to atherosclerosis and thrombosis by promoting leukocyte adhesion, platelet activation, and oxidative stress. This imbalance disrupts the equilibrium between vasodilatory and vasoconstrictive factors. Normally, ECs maintain vascular tone by producing vasodilators such as prostacyclin (PGI_2_), NO, and EDHF. However, in the presence of metabolic stressors such as elevated FFAs and oxidative stress, these vasodilatory signals are suppressed, while vasoconstrictive mediators including angiotensin II (Ang II) and prostaglandin H_2_ (PGH_2_) are upregulated, further exacerbating vascular dysfunction and hypertension [82]. A key feature of atherosclerotic progression is the upregulation of adhesion molecules on ECs, facilitating monocyte and leukocyte recruitment into the vascular wall. ECs express several adhesion proteins, including selectins, ICAMs, and VCAM-1, which play essential roles in early atherogenesis. These adhesion molecules mediate the firm attachment and transmigration of inflammatory cells into the intima, where they differentiate into macrophages and eventually transform into foam cells, contributing to plaque development and instability [83]. The mechanisms of fatty acid (FA) uptake by ECs remain incompletely understood, but evidence suggests a combination of protein-mediated transport and passive diffusion. Once inside the cytoplasm, FA metabolism depends on chain length, saturation, and double-bond positioning. In mitochondria, FFAs undergo β-oxidation, generating ATP through the tricarboxylic acid (TCA) cycle to support endothelial function [84,85]. Elevated circulating FFAs can impair endothelial function by promoting inflammation and increasing endothelial permeability [86]. Increased FFA levels in the blood also inhibit insulin-mediated NO generation and diminish peripheral blood flow [87]. FFAs promote these effects by two distinct mechanisms: decreasing the tyrosine phosphorylation of IRS-1/2 and suppressing the PI3K/Akt pathway, which regulates insulin-stimulated glucose uptake and NO generation by eNOS [86]. This suggests that FFAs specifically inhibit NO production by ECs. In summary, endothelial dysfunction in diabetes is multifactorial, driven by lipid abnormalities, insulin resistance, chronic inflammation, and oxidative stress. The interaction between FFAs, adhesion molecules, and impaired insulin signaling accelerates atherosclerosis and increases the risk of thrombotic complications. Further research is needed to identify therapeutic strategies that effectively target FFA-induced endothelial dysfunction, with the goal of reducing cardiovascular risk in diabetic patients.

## 3. Targeting Mitochondrial Dysfunction in Endothelial Cells: Therapeutic Potential of BAM15 and Mitochondrial Uncoupling in Diabetes-Induced Atherosclerosis

### 3.1. Metabolic Reprogramming in Endothelial Cells: Implications for Vascular Health

ECs rely on three primary metabolic substrates—glucose, fatty acids (FAs), and amino acids (AAs)—to generate ATP and maintain cellular function. Among these, glycolysis is the dominant energy-producing pathway, even in the presence of oxygen. Unlike many other metabolically active cells that favor oxidative phosphorylation (OXPHOS), ECs depend on glycolysis to minimize ROS production. This adaptation is crucial for maintaining vascular homeostasis, as excessive ROS can cause endothelial dysfunction and promote the progression of atherosclerosis and other CVDs [88,89]. Beyond its role in energy production, glycolysis also plays a vital function in endothelial proliferation, migration, and angiogenesis. The rapid ATP production from glycolysis allows ECs to efficiently respond to proangiogenic signals, facilitating vascular remodeling and new blood vessel formation [90]. ECs also use glutamine to maintain proliferation and vascular expansion. Glutamine, the most abundant circulating nonessential amino acid (NEAA), can contribute 30% of the TCA carbons, comparable to glycolysis and FAO-derived carbon [91]. When glutamine or arginine levels are low, ECs are more sensitive to ROS-induced damage during proliferation and migration [92]. FA metabolism entails a number of mechanisms such as FA absorption and storage, transport, oxidation, and synthesis. ECs can metabolize and synthesis FAs to maintain vascular homeostasis [91]. FA metabolism is essential for the proper function of energy-demanding organs such as the heart, skeletal muscle, and adipose tissue. FAs are carboxylic acids consisting of long aliphatic chains with a methyl group at one end and a carboxyl group at the other. They are classified by chain length into short-chain (SCFAs), medium-chain (MCFAs), and long-chain fatty acids (LCFAs). Additionally, FAs are categorized based on the presence of double bonds: saturated fatty acids (SFAs) contain no double bonds, monounsaturated fatty acids (MUFAs) contain one, and polyunsaturated fatty acids (PUFAs) contain two or more [79,86]. FA metabolism consists of several processes. FA uptake and transport by ECs are critical to many cellular activities, including membrane formation, intracellular signal transduction, ATP generation, protein post-translational modifications, and metabolic gene transcriptional control in these high-energy-demanding organs [93,94]. Lipids circulate in the mammalian system as nonesterified free fatty acids in the blood, including MCFAs (with 6-12 carbons) and SCFAs (with 6 carbons), but primarily as esterified FAs, including LCFAs (with 12-20 carbons) [95]. LCFAs are transported in the bloodstream as triglyceride (TG)-rich lipoproteins, phospholipids, and cholesteryl esters in lipoproteins [96]. LCFA uptake requires transport across the EC barrier, a process regulated by several membrane-associated proteins, including lipoprotein lipase, glycosylphosphatidylinositol-anchored high-density lipoprotein-binding protein 1, CD36, fatty acid transport proteins (FATPs), and fatty acid-binding proteins (FABPs).

The primary FA transporters—CD36, FATPs, and FABPs—will be discussed in detail in the following sections. CD36, expressed in ECs, platelets, cardiac muscle, and skeletal muscle, plays a key role in FA uptake and is involved in various cell signaling pathways [97,98]. Studies using EC-specific CD36 knockout (CD36-KO) mice have shown reduced FA uptake in the heart, skeletal muscle, and brown adipose tissue (BAT), highlighting the critical role of endothelial CD36 in facilitating FA transport into parenchymal tissues [91,99]. It has also been demonstrated that in mice, the EC-specific ablation of CD36/LDLR enhanced glucose clearance and reduced atherosclerosis [100]. FATPs are transmembrane proteins that facilitate and promote the absorption of LCFAs into cells. Their expression pattern is tissue-specific [101]. FABP4, also known as A-FABP and aP2, is the most investigated FABP in ECs when compared to other FABPs [102,103]. FABPs can directly bind free LCFAs and enhance their transport to various intracellular locations for oxidation. FABPs are members of a family that includes at least 13 intracellular FA-handling proteins. FABP4 and FABP5 share a 55% amino acid sequence similarity and exhibit a mainly microvascular expression pattern in both microvascular and macrovascular ECs [91,104]. FABP4 and FABP5 deletion (DKO) mice revealed a striking phenotype associated with protection against obesity, insulin resistance, atherosclerosis, and fatty liver disease [105].

### 3.2. Dysregulated Fatty Acid Metabolism in Endothelial Dysfunction and Atherosclerosis

FAs are commonly stored as TGs, which consist of three FA molecules linked to a glycerol backbone. These stored lipids serve as a major energy reservoir in adipose tissue, releasing FFAs during periods of energy demand. When dietary fat intake exceeds immediate metabolic requirements, excess FAs are esterified into TGs and stored primarily in adipocytes [106]. Metabolic disorders such as obesity and diabetes disrupt lipid metabolism, resulting in elevated circulating levels of SFAs and triglyceride-rich lipoproteins. This imbalance contributes to endothelial dysfunction and accelerates atherosclerosis. In obesity, impaired adipose tissue function leads to excessive lipolysis and increased FFA release into circulation. These FFAs are taken up by peripheral tissues for oxidation or lipid synthesis. However, excessive lipid accumulation in non-adipose tissues, including the endothelium, induces lipotoxicity, oxidative stress, and pro-inflammatory signaling—all key drivers of vascular dysfunction [107]. Glucose and lipid metabolism are tightly interconnected at multiple regulatory levels. The most prominent clinical expression of this association is diabetic dyslipidemia, which is marked by high levels of TGs in VLDL and low levels of HDL cholesterol [108]. Because this type of dyslipidemia is a valuable predictor of CVD development, examining the impact of body fat distribution on FFA metabolism and dyslipidemia is crucial for understanding how diabetes and obesity increase the risk of CVD [109].

FAO accounts for a limited amount of the total ATP production in ECs [91,110]. FAO contributes up to 40% of ATP production in ECs. FAO-derived carbons fuel the TCA cycle, supporting the synthesis of aspartate—a key precursor for deoxyribonucleotide (dNTP) production during EC proliferation [91,111]. Although the role of mitochondrial-dependent FAO in atherosclerosis is not fully understood, FAO is essential for maintaining metabolic homeostasis. The carnitine palmitoyltransferase (CPT) system, comprising CPT1 and CPT2, regulates the mitochondrial import and oxidation of LCFAs [110,111]. CPT1 and CPT2 are involved in transporting LCFAs into the mitochondria [112]. FAO is considered atheroprotective, suggesting that enhancing FAO may help reduce atherosclerosis progression [113]. EC activity is known to be dependent on FA production. ECs lacking FA production have insufficient migratory capacity, and angiogenesis is inhibited [114,115]. The inhibition of CPT1A has shown that FAO is a key regulator of endothelial permeability in vitro and vascular stability in vivo [116]. Silencing CPT1A, the rate-limiting enzyme for FAO, affects EC sprouting in vitro and in vivo due to a reduction in the dNTP pool [111]. Surprisingly, quiescent ECs express a higher quantity of FAO genes to maintain the TCA cycle for redox equilibrium via NADPH regeneration [115]. Mice lacking EC-specific CPT1A induce EC dysfunction by increasing oxidative stress [117]. In culture, macrophages generated from CPT1a and CPT2 M-KO mice show defective FAO, increased expression of the CD36 scavenger receptor, increased absorption of ox-LDL, and heightened transformation into cholesterol-rich foam cells [113]. The inhibition of CPT1A increases ROS production, leading to the reduced expression of antifibrinolytic genes, heightened vascular permeability, and enhanced leukocyte adhesion and migration [115]. Beyond ECs, dysregulated FA metabolism also impacts macrophage function within atherosclerotic lesions. Macrophages from CPT1A- and CPT2-deficient mice display impaired FAO, upregulated expression of the scavenger receptor CD36, and increased uptake of oxLDL, promoting foam cell formation. CD36 plays a critical role in mediating the uptake of oxLDL and contributing to macrophage lipid overload, leading to the formation of cholesterol-rich foam cells and the progression of atherosclerotic plaques [118]. The inhibition of CPT1A further augments vascular permeability and inflammatory cell recruitment while activating pro-inflammatory and pro-thrombotic pathways. Impaired FAO also elevates ROS levels, exacerbating endothelial dysfunction and vascular inflammation.

### 3.3. Angiogenic Metabolism in Endothelial Cells: Balancing Energy Demand and Vascular Growth

The endothelium, which lines the inner surface of blood vessels, plays a crucial role in regulating oxygen and nutrient supply to tissues. Under normal physiological conditions, ECs remain quiescent, forming a stable barrier. However, in response to hypoxia or nutrient deprivation, quiescent ECs are activated to initiate angiogenesis—the process of forming new blood vessels from pre-existing ones [119,120]. This dynamic process is tightly regulated by metabolic and signaling cues from the surrounding microenvironment. Various proangiogenic factors, such as vascular endothelial growth factor (VEGF), are secreted by oxygen-deprived tissues to stimulate ECs. In response, ECs undergo distinct morphological and functional changes, leading to vascular sprouting and network expansion [90,121]. During angiogenesis, leading ECs differentiate into tip cells, which extend filopodia toward angiogenic cues and guide sprout direction. Adjacent stalk cells proliferate to elongate and support the growing vessel. ECs dynamically switch between tip and stalk phenotypes. As sprouts extend, tip cells from neighboring vessels fuse, forming functional loops. Once oxygen and nutrient needs are met, proangiogenic signals wane, and ECs revert to quiescence. Pericytes are recruited to stabilize the vessel, and basement membrane deposition provides structural support. Mature phalanx cells then maintain vessel integrity and blood flow [90,122,123,124].

ECs exhibit distinct metabolic activity compared to other differentiating cells. During angiogenesis, ECs primarily generate ATP through aerobic glycolysis rather than OXPHOS [89]. Proangiogenic factors like VEGF enhance glycolysis by increasing glucose uptake and upregulating glycolytic enzymes such as lactate dehydrogenase A (LDH-A) and key activators like phosphofructokinase-2/fructose-2,6-bisphosphatase 3 (PFKFB3) [125,126]. The inhibition or silencing of PFKFB3 suppresses sprouting and tip/stalk cell formation, whereas its overexpression promotes glycolysis and favors a tip cell phenotype [127,128]. Aside from the critical involvement of glycolysis in tip and stalk cell function during vascular sprouting, recent data have revealed the significance of the previously neglected FAO pathway during angiogenic processes [91,129]. FAs are another source of energy. They are either passively diffused from blood or transported by FA transporters into the cell to fuel the TCA cycle. The oxidation of FAs sustains vessel growth and development. Normal stalk cell functions are dependent on FAO [91,120]. The genetic or pharmacological inhibition of CPT1, the rate-limiting enzyme in FAO, impairs EC differentiation, proliferation, and barrier integrity. FAO supports dNTP synthesis, essential for EC proliferation during sprouting angiogenesis. In addition to glycolysis and FAO, ECs rely on glutamine as a key nutrient [89,91,120]. Glutamine, the most abundant non-essential amino acid in plasma, enters cells via solute carrier (SLC) family transporters. It is converted by glutaminase into glutamate and ammonia and then by glutamate dehydrogenase into α-ketoglutarate, which fuels the TCA cycle via anaplerosis [130,131]. These findings highlight the central role of glycolysis in EC activation and angiogenesis. The metabolic flexibility of ECs is essential for angiogenesis, enabling them to adapt energy sources based on environmental cues. Glycolysis supports rapid ATP production and tip cell migration, while FAO sustains stalk cell proliferation, and glutamine metabolism drives biosynthesis. The dysregulation of these pathways contributes to vascular diseases such as atherosclerosis, diabetic retinopathy, and tumor angiogenesis. Understanding EC metabolism offers novel therapeutic opportunities. Targeting metabolic regulators like PFKFB3, CPT1A, and GLS may provide effective strategies to control pathological angiogenesis.

### 3.4. Lipid-Lowering Therapeutics: Current Strategies and Their Limitations in Diabetes-Associated Atherosclerosis

Several clinical trials have explored strategies to improve EC function in diabetic patients to slow atherosclerosis progression. While hypolipidemic drugs are widely used, their specific effects on EC function in diabetes remain unclear. These drugs reduce the blood levels of cholesterol and triglyceride-carrying lipoproteins. Common classes include statins, fibrates, bile acid sequestrants, nicotinic acid, and acipimox. Statins (e.g., atorvastatin, cerivastatin, fluvastatin, pravastatin, simvastatin) lower cholesterol by inhibiting HMG-CoA reductase, a key enzyme in cholesterol biosynthesis [132,133,134]. Several clinical trials have shown that statin therapy not only lowers serum cholesterol levels in hypercholesterolemic patients but also significantly lowers the risk of CVD [135,136]. Fibrates (e.g., bezafibrate, ciprofibrate, clofibrate, fenofibrate, gemfibrozil) lower plasma TG and enhance the breakdown of LDL-C. They promote FA uptake, conversion to acyl-CoA derivatives, and catabolism via FAO pathways. This, combined with reduced FA and TG synthesis, leads to decreased VLDL formation [137]. Bile acid sequestrants (cholestyramine and colestipol) bind to bile acids and reduce fat absorption, increasing the quantity of fat expelled in the feces and reducing plasma cholesterol levels. Bile acid sequestrants (BASs) lower plasma LDL cholesterol by disrupting enterohepatic circulation, prompting the liver to convert more cholesterol into bile acids. This increases bile acid excretion and reduces overall cholesterol levels. BASs are effective cholesterol-lowering agents with minimal adverse effects [138,139]. They can be used in conjunction with statins if high-dose statins fail to attain the desired LDL-C levels. Nicotinic acids (acipimox) are a vitamin B complex that lowers cholesterol and TG levels in the blood while increasing high-density lipoprotein cholesterol (HDL-C). Nicotinic acid (niacin), a form of vitamin B3, improves lipid profiles by reducing fat breakdown in adipose tissue. As a therapeutic agent, it lowers triglycerides and LDL cholesterol while raising HDL cholesterol [140,141,142]. Although lipid-lowering therapies play a crucial role in managing diabetes-associated dyslipidemia and reducing cardiovascular risk, their efficacy in fully restoring EC function remains unclear. Diabetes is characterized by a complex interplay of metabolic dysfunction, oxidative stress, and chronic inflammation, which contribute to endothelial dysfunction and atherosclerosis beyond lipid abnormalities. Therefore, while current lipid-lowering strategies effectively reduce circulating lipid levels, their ability to directly improve EC health and prevent diabetic vasculopathy warrants further investigation. Future therapeutic approaches may need to combine lipid modulation with targeted interventions that address endothelial metabolism, oxidative stress, and inflammation to achieve comprehensive vascular protection in diabetic patients.

### 3.5. Mitochondrial Uncoupling as a Novel Therapeutic Approach: Potential of BAM15 in Restoring Endothelial Function

Mitochondrial uncoupling has recently emerged as a promising therapeutic strategy for metabolic disorders, including diabetes and obesity. This process involves the dissociation of mitochondrial respiration from ATP synthesis, allowing the electron transport chain to function without generating excessive ATP. Mitochondrial uncoupling reduces membrane potential, thereby lowering oxidative stress and enhancing cellular metabolism—offering a promising strategy to restore endothelial function in diabetes-related vascular complications [143]. Mitochondrial uncouplers are emerging as therapeutic agents for metabolic disorders, including diabetes and obesity, as they can enhance mitochondrial function and promote lipid oxidation. Recent studies have highlighted their potential to improve metabolic balance and restore endothelial function in the context of diabetes-induced vascular dysfunction [118,144,145]. A variety of factors can cause mitochondrial uncoupling, and uncoupling mitochondrial respiration lowers the coupling between electron transport and OXPHOS processes, inhibiting ATP synthesis without affecting the respiratory chain or ATP synthase. Mitochondrial uncoupling can be induced through various mechanisms, including uncoupling proteins (UCPs), adenine nucleotide translocases (ANTs), or synthetic uncouplers. Common synthetic agents used in vitro include BAM15, FCCP, CCCP, 2,4-dinitrophenol (DNP), and FFAs. Among them, FCCP (carbonyl cyanide p-trifluoro-methoxyphenyl hydrazone) and CCCP (carbonyl cyanide-3-chlorophenylhydrazone) are the most widely used in basic research [146,147]. Mitochondrial uncoupling by DNP causes weight reduction in people. FCCP and CCCP are the most commonly used in basic research due to their potent ability to disrupt mitochondrial membrane potential. Historically, DNP was utilized as an anti-obesity drug in the 1930s due to its capacity to enhance the metabolic rate and promote weight loss. However, its clinical application was discontinued due to severe side effects, including life-threatening hyperthermia, leading to its ban by the FDA. In contrast, BAM15, a novel mitochondrial uncoupler, shows improved safety and therapeutic promise in preclinical studies (Table 1). It enhances energy expenditure, improves glucose metabolism, and promotes lipid oxidation with minimal off-target effects. In high-fat diet (HFD)-induced obese mice, BAM15 significantly reduced adiposity while preserving systemic metabolic homeostasis, distinguishing it from earlier uncouplers with poor safety profiles [143,145,148].

**Figure 3:**
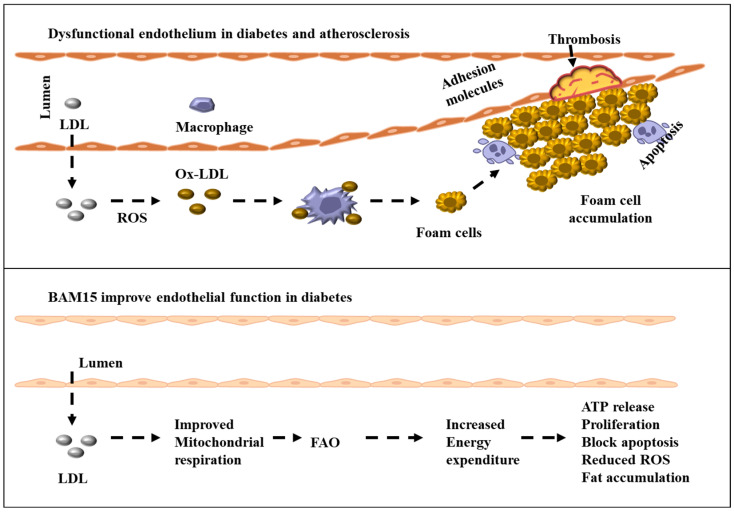
Schematic illustration showing the potential protective effects of BAM15 against atherosclerosis by enhancing EC function under hyperglycemic conditions. BAM15 mitigates mitochondrial damage by regulating membrane potential, reducing ROS, and inhibiting apoptosis. Through mild mitochondrial uncoupling, BAM15 increases energy expenditure, promotes FAO, and reduces intracellular lipid accumulation. These effects suggest BAM15 may serve as an effective therapeutic strategy for diabetes-associated atherosclerosis.

Mitochondrial uncoupling increases oxygen consumption in the diabetic heart and may boost FAO rates even further [158,159]. BAM15 increases mitochondrial function and glycemic management via regulating insulin signaling [143,148]. We found that low-dose BAM15-induced mild mitochondrial uncoupling enhances EC survival under hyperglycemic conditions, outperforming insulin in promoting cell viability. Our unpublished data indicate that BAM15 improves mitochondrial complex I and IV activity and increases ATP production in ECs. Furthermore, gene expression analysis revealed that hyperglycemia suppressed FAO-related genes in ECs, while BAM15 treatment restored their expression. These findings underscore the therapeutic potential of BAM15 in protecting ECs by modulating mitochondrial metabolism and improving energy homeostasis. It remains unclear whether these effects are solely due to a glycolytic shift or if other mitochondrial metabolites contribute to this compensatory response. To explore this mechanism, we assessed the expression of genes regulating FAO and found that hyperglycemia suppressed FAO-related gene expression in ECs. In contrast, BAM15 restored their expression under hyperglycemic conditions (unpublished data). These findings highlight BAM15 as a promising therapeutic strategy for diabetes-induced endothelial dysfunction. By modulating mitochondrial metabolism, BAM15 enhances energy balance and protects ECs from hyperglycemia-driven damage. Further studies are warranted to evaluate its long-term efficacy and clinical relevance in managing diabetes-related vascular complications. An overview of the proposed mechanisms by which BAM15 exerts its protective effects on ECs under hyperglycemic conditions is illustrated in Figure 3. This schematic summarizes how BAM15 reduces mitochondrial damage, lowers ROS production, inhibits apoptosis, and promotes FAO, collectively contributing to vascular protection in diabetes-associated atherosclerosis.

## 4. Conclusions

Despite advances in our understanding of diabetes-associated atherosclerosis, the precise mechanisms linking hyperglycemia, dyslipidemia, and endothelial dysfunction remain to be fully elucidated. Endothelial dysfunction plays a central role in the progression of atherosclerosis, particularly under diabetic conditions where metabolic imbalance exacerbates vascular injury. Although lipid-lowering and glucose-lowering agents have improved patient outcomes, many current therapies fall short of targeting the multifaceted pathology of diabetes-related vascular disease and are often limited by adverse effects. Mitochondrial dysfunction is increasingly recognized as a critical contributor to endothelial impairment and atherosclerosis. Among emerging strategies, mitochondrial uncouplers offer a novel approach by promoting FAO, reducing oxidative stress, and enhancing energy expenditure. In particular, BAM15 has shown preclinical promise in restoring mitochondrial function, improving glucose and lipid metabolism, and protecting ECs from hyperglycemia-induced damage.

Overall, targeting mitochondrial metabolism via mild uncoupling may represent a promising avenue for the treatment of diabetes-associated atherosclerosis. Future studies should further elucidate the molecular mechanisms underlying these effects and evaluate the long-term safety and efficacy of BAM15 in relevant preclinical and clinical settings. While preparing this review, we encountered certain limitations, including the lack of available clinical data on BAM15 and the reliance on preclinical rodent models. Additionally, given the rapid pace of emerging research in this area, there may be recent developments not captured at the time of manuscript preparation. Nonetheless, we have strived to provide a comprehensive and up-to-date synthesis of current knowledge while highlighting key areas for future investigation.

## Figures and Tables

**Table 1:** Overview of preclinical investigations on BAM15: experimental models, dosing, and biological effects.

Author (Year)	Model	BAM15 Dose and Duration	Key Findings	Functional Outcomes	Ref.
Stephanie J Alexopoulos et al. (2020)	Diet-induced obesity in male C57BL/6J mice	0.05–0.15% *w*/*w* in diet for 8 days (prevention)	BAM15 is orally bioavailable; reduces body fat, hepatic fat, and inflammatory lipids; improves insulin sensitivity without affecting food intake or lean mass; increases nutrient oxidation	BAM15 is a unique mitochondrial uncoupler that effectively prevents and reverses diet-induced obesity without reducing food intake or compromising lean body mass	[143]
Christopher L Axelrod et al. (2020)	4-week-old male C57BL/6J mice; high-fat diet (HFD)	0.1% *w*/*w* in diet (chronic exposure)	BAM15 increased energy expenditure, improved glucose and lipid metabolism, enhanced AMPK activation, and improved insulin sensitivity	Protection against diet-induced obesity and improved glycemic control independent of weight loss	[148]
Cong Phi Dang et al. (2021)	LPS-induced systemic inflammation in mice	1 mg/kg i.p., 3 h before LPS (4 mg/kg)	Reduced serum and tissue pro-inflammatory cytokines; enhanced hepatic AMPK activation; reduced inflammatory monocyte infiltration in liver	Attenuated organ injury (liver enzymes, creatinine); improved inflammatory and metabolic profile in sepsis	[149]
Wagner S Dantas et al. (2022)	Sarcopenic obesity in aged male C57BL/6J mice (80 weeks)	0.1% *w*/*w* in high-fat diet for 10 weeks	BAM15 reduced body weight and fat mass, enhanced energy expenditure, increased skeletal muscle mass and strength, improved mitochondrial quality control, and reduced ER stress and apoptosis	Attenuated sarcopenic obesity; improved muscle function and metabolic health in aged mice	[150]
Injeong Cho et al. (2022)	Caenorhabditis elegans (wild-type and ucp-4 mutants)	50 µM (treatment during aging)	BAM15 treatment reduced mechanosensory neuronal defects and preserved touch responses and short-term memory in aging nematodes; it also extended the lifespan of both wild-type and ucp-4 mutants	Reduced neurodegeneration and extended lifespan in *C. elegans*	[151]
Pratsanee Hiengrach et al. (2022)	Male C57BL/6J mice, cecal ligation and puncture (CLP) sepsis model	5 mg/kg (administered before and 6 h post-CLP surgery)	BAM15 attenuated sepsis by reducing organ damage, systemic inflammation, mitochondrial injury, and neuronal miR370-3p upregulation; it also reduced blood–brain barrier damage and apoptosis in spleen and brain	Reduced mitochondrial injury, decreased systemic inflammation, reduced neuronal miR370-3p, improved blood–brain barrier integrity, and alleviated brain injury and encephalopathy	[152]
Kanyarat Udompornpitak et al. (2023)	C57BL/6 mice with LPS-induced sepsis	BAM15: 2 mg/kg i.p.; BAM15 particles: 2 mg/kg i.p. (before LPS)	BAM15 particles specifically targeted macrophages, reduced inflammation, and improved mitochondrial activity; BAM15 and BAM15 particles reduced LPS-induced liver injury and sepsis severity	Reduced inflammation and liver injury and improved mitochondrial activity in macrophages	[153]
Naoko Tsuji et al. (2023)	Male 6-week-old mice, CLP-induced sepsis	1 mg/kg i.p. at 0, 6, or 12 h post-CLP + antibiotics/fluids	Reduced mortality, kidney damage, and splenic apoptosis; decreased plasma/urine mtDNA and mtROS in tubule cells	BAM15 prevented neutrophil apoptosis and mtDNA release; mtDNA injection reversed effects, linking mtROS and mtDNA with sepsis pathology	[154]
Sing-Young Chen et al. (2024)	Male C57BL/6J mice fed a high-fat Western diet (diet-induced obesity)	0.05% (*w*/*w*) in food; 4 weeks of treatment	Combining BAM15 with semaglutide (low or high dose) improved body fat reduction and glucose control while preventing lean mass loss and liver TG accumulation	Improved weight loss and glucose homeostasis and reduced body fat without lean mass loss	[155]
Analisa L Taylor et al. (2024)	Drosophila melanogaster on normal diet (ND) or HFD	0.036% (*w*/*w*) supplemented with diet (ND or HFD) for the duration of lifespan study	BAM15 extended lifespan by 9% on ND and 25% on HFD, improved locomotor activity, enhanced oxidative phosphorylation, and upregulated mitochondrial function and antioxidant defense	Extended lifespan, enhanced locomotor function, and improved mitochondrial redox capacity and fitness	[156]
Minghui Ma et al. (2025)	ApoE (−/−) mice fed a Western diet to establish atherosclerosis model	85 mg/kg/day (oral, 6 times a week)	BAM15 inhibited atherosclerosis in WD-fed ApoE (−/−) mice; improved hyperlipidemia; reduced ALT, AST, liver TC, and TG levels; inhibited macrophage invasion and lipid accumulation in vitro	Reduced atherosclerosis progression; improved lipid profile and macrophage function	[157]

## Data Availability

The data used to support the findings of this study are included in the article.
